# Adherence and Radiological Outcomes in Braced Adolescents with Idiopathic Scoliosis: A Real-World Study Using Thermal Sensors

**DOI:** 10.3390/jcm14248648

**Published:** 2025-12-06

**Authors:** Samra Pjanić, Fabio Zaina, Nikola Jevtić, Filip Golić, Vanja Dimitrijević, Bojan Rašković, Snježana Novaković-Bursać, Dragana Bojinović-Rodić, Goran Talić

**Affiliations:** 1 Institute for Physical Medicine, Rehabilitation and Orthopedic Surgery “Dr Miroslav Zotovic”, 78000 Banja Luka, Bosnia and Herzegovina; samra.pjanic@zotovicbl.com (S.P.); filip.golic@zotovicbl.com (F.G.); snjezana.nb@ms.zotovicbl.org (S.N.-B.); bojinovic.rodicd@ms.zotovicbl.org (D.B.-R.); kancelarija.direktora@ms.zotovicbl.org (G.T.); 2 ISICO (Italian Scientific Spine Institute), 20141 Milan, Italy; fabio.zaina@isico.it; 3 Scolio Centar, 21000 Novi Sad, Serbia; njevticns@gmail.com; 4 Faculty of Sport and Physical Education, University of Novi Sad, 21000 Novi Sad, Serbia; vanja.dimitrijevic@fsfvns.edu.rs; 5 Functionally Aware Motoric Activity (FAMA) Center, 21000 Novi Sad, Serbia; 6 Performance Zone, 21000 Novi Sad, Serbia; 7 Faculty of Medicine, University of Banja Luka, 78000 Banja Luka, Bosnia and Herzegovina

**Keywords:** idiopathic scoliosis, adolescent idiopathic scoliosis, brace treatment, patient adherence, thermal sensors, objective monitoring, cobb angle

## Abstract

**Background**: Bracing is an effective treatment for idiopathic scoliosis (IS), and Adherence is one of the most important factors influencing outcomes. However, evidence on the effectiveness of objective monitoring using thermal sensors in everyday clinical settings remains limited. Objective: To evaluate the impact of Adherence and brace-wearing consistency on short-term outcomes in IS patients during growth in a real clinical setting. **Methods**: This retrospective cohort study included 114 patients (100 with adolescent idiopathic scoliosis (AIS) and 14 with juvenile idiopathic scoliosis (JIS)) treated with a three-dimensional (3D) rigid brace equipped with a thermal sensor. Adherence was calculated as the ratio of actual to prescribed wear time; consistency was defined using the interquartile range (IQR) of daily wear time (IQR ≤ 1 h = consistent). Cobb angle was measured at baseline, in-brace, and after 6 months. Logistic regression, receiver-operating characteristic (ROC) analysis, and general linear model (GLM) analyses were performed, supported by sensitivity analyses, to assess model robustness. **Results**: Mean Adherence was 85.3 ± 18.9%; high Adherence was observed in 71.4% of JIS and 57.0% of AIS patients, while 19.3% showed high consistency. In-brace correction averaged 52.5 ± 23.2% (68.1% JIS vs. 50.3% AIS; *p* = 0.007). At 6 months, 55% improved, 42% were stable, and 3% worsened. Adherence was the only significant predictor of consistency (OR = 1.511; 95% CI 1.181–1.933; *p* = 0.001). The ROC analysis showed excellent discriminative ability (Area Under the Curve (AUC) = 0.908). Adherence category (*p* = 0.041) and Risser stage (*p* = 0.041) were significant predictors of short-term outcome. Conclusions: Adherence and brace-wearing consistency are key predictors of short-term bracing outcomes in IS. Objective monitoring with thermal sensors enables precise tracking, improves patient engagement, and supports individualised treatment.

## 1. Introduction

Bracing proved effective in stopping scoliosis progression and, eventually, in improving the Cobb angle, trunk deformity, and aesthetic asymmetries [1,2].

Among the main factors predicting brace success are the three-dimensional in-brace correction, evaluated clinically and radiographically [3], and the actual daily dosage [4,5]. The higher the dosage, the greater the likelihood of a positive outcome [6]. Nonetheless, treatment adherence can be challenging [7]. Adolescence is a challenging time, and young patients may struggle to follow their treating physicians’ instructions. Specific subjective factors, such as high self-esteem, above-average peer relationships, and poor brace-specific attitudes, are associated with lower brace adherence [8]. It is clear that Adherence goes beyond the brace itself [5], and for this reason, recommendations were made by The International Society on Scoliosis Orthopaedic and Rehabilitation Treatment (SOSORT) [9].

For a couple of decades, tools have been developed to monitor the objective dosage and thus treatment adherence in different fields, such as orthopaedic and rehabilitation treatment [10]. For trunk orthoses, strap tension and pressure sensors have been developed and tested first [11,12,13], while thermal sensors have gained popularity more recently [14,15].

Objective monitoring of daily brace-wearing time with such tools can allow for counselling patients, supporting them in increasing their Adherence and thus improving outcomes [16]. A small RCT showed that awareness of objective monitoring correlated with increased adherence [17]. Despite this positive feedback and the undeniable benefits of objective monitoring, a wide clinical application of sensors remains limited, and most published papers include small samples followed for short periods, primarily for research purposes.

At our Institute, since 2022, we have started applying thermal sensors to all braces to support our patients in their treatment management. This paper aims to provide short-term Adherence and Consistency data for a comprehensive rehabilitation treatment involving bracing and exercise, objectively measured with thermal sensors in a real clinical setting. The secondary aim is to document the radiological results, namely the changes in the Cobb angle over 6 months. We hypothesise that results will be directly related to the Adherence to the prescribed dosage.

## 2. Materials and Methods

### 2.1. Study Design

This is a retrospective study based on prospectively collected data from the Institute for Physical Medicine, Rehabilitation, and Orthopaedic Surgery’s “Dr Miroslav Zotovic” database between January 2022 and September 2024. The institutional ethics committee approved the study on 11 December 2024. (approval number: 21-01-7947-1/24).

Inclusion criteria: We included children with IS aged 18 years or younger of both genders, who were prescribed a first brace by the Institute’s Scoliosis Team and had a thermal sensor applied. The patients underwent comprehensive treatment with a 3D rigid brace and PSSE in accordance with the SOSORT guidelines.

Exclusion criteria: secondary scoliosis and surgically treated patients.

All patients were prescribed full-time bracing (18–23 h per day) using a 3D rigid brace fabricated individually and fitted by certified orthotists. According to the new classification, the braces were a TLSO, rigid detorsion, three-dimensional, Monocot, ventral closure brace (Cheneau) and a TLSO, very rigid, push-up, three-dimensional, bivalve, ventral closure brace (Sforzesco) [18]. Follow-up visits were scheduled regularly to ensure proper fit and monitor compliance.

### 2.2. Protocol

A standardised protocol was applied, consisting of full-spine radiographs in AP projection: at baseline, in the brace after 1 month to assess brace quality, and out of brace after 6 months to assess short-term outcome. Before taking an out-of-brace radiograph, patients were instructed to remove the brace for the same number of hours they would normally spend without wearing it within 24 h (e.g., 20 h of wear time—4 h out of brace before the X-rays).

Family history of scoliosis was included in the anamnestic data. Clinical parameters included ATR (Angle of Trunk Rotation) at the level of the primary curve. Radiological parameters included the Cobb angle of the primary curve, the location of the primary curve, apical vertebral rotation (measured with the Raimondi method), and the Risser sign. The primary curve was defined as the one with the greatest structural changes (the most laterally displaced and most horizontally oriented) [19]. Compensatory curves were taken into consideration during the interpretation of the results, ensuring that the correction of the primary curves did not lead to the development or worsening of compensatory curves in other spinal segments, nor that the correction in the frontal plane caused deterioration in the other two planes (transversal and sagittal).

**Figure 1:**
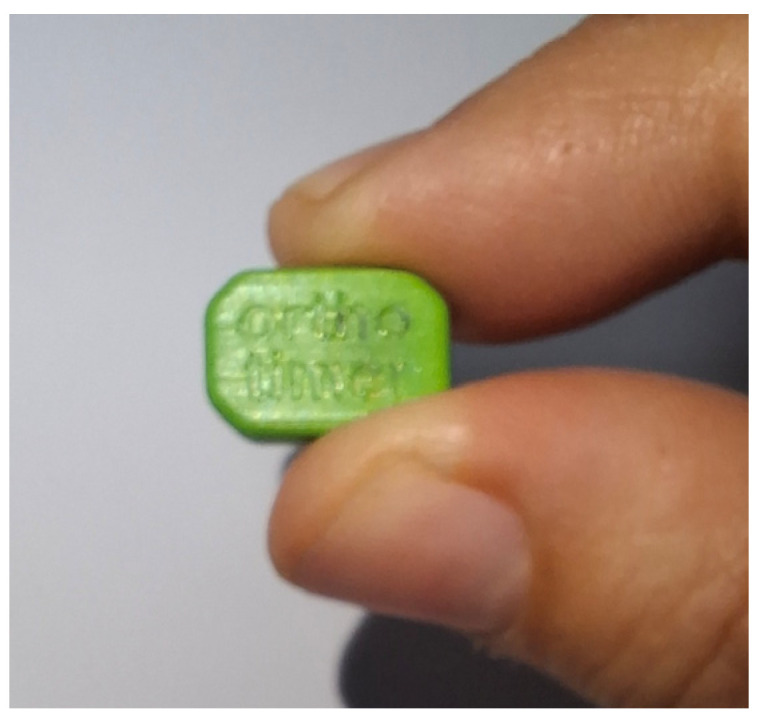
OrthoTimer^®^ thermal sensor positioned inside the brace.

Objective Adherence was assessed using a temperature-sensitive sensor embedded in the brace (OrthoTimer^®^, Rollerwerk-Medical/Orthotimer, Balingen, Germany). The sensor is a small, lightweight device placed on the inner surface of the brace to capture the temperature difference between body contact and ambient conditions. A photograph of the sensor and its positioning within the brace is shown in Figure 1.

Objective Adherence was assessed using a temperature-sensitive sensor embedded in the brace (OrthoTimer^®^). The device has been used in previous research on different types of upper- and lower-limb braces [20,21,22,23]. It continuously recorded the temperature inside the brace at regular 15 min intervals, allowing for accurate determination of actual brace-wear time.

**Figure 2:**
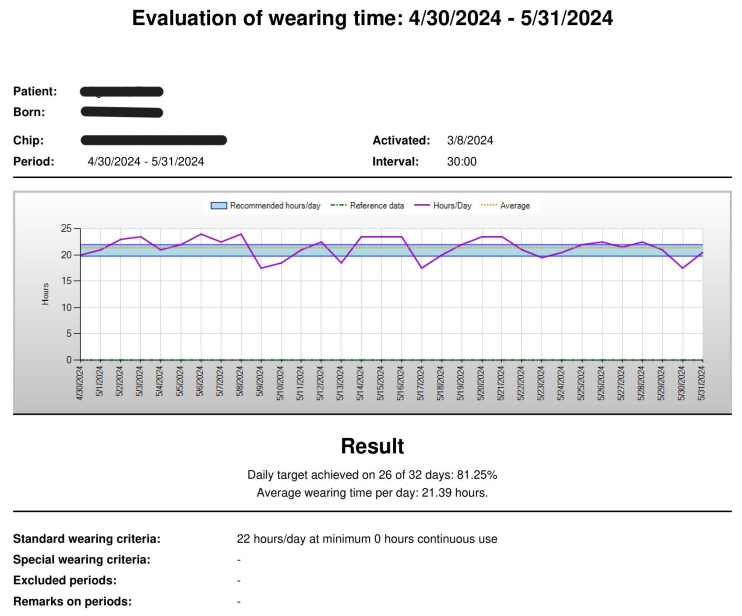
Example of daily brace-wear time readout generated by the thermal sensor.

Raw temperature data were downloaded during each follow-up visit and analysed using the manufacturer’s proprietary software, which automatically converts temperature curves into daily wear-time patterns. An example of the sensor-generated wear-time output is presented in Figure 2.

### 2.3. Outcomes

The patients were divided into groups based on age (JIS and AIS), treatment Adherence, consistency, and short-term outcome.

For the main outcomes, Adherence and consistency, we used the same definitions and methodology as Donzelli 2018 [24]. We defined “Adherence” as the ratio of the average daily brace wear time to the recommended daily wear time (in hours).

To define consistency in brace wear, we used a consistency index, calculated as the interquartile range of daily thermosensor-recorded brace-wear time. Patients with a consistency index of 1 or less were classified as consistent patients, while those with a consistency index greater than one were classified as inconsistent patients [24].

To identify subjects with greater data scattering and distinguish them from those with more homogeneous data, measures of variability were applied. The abnormal distribution of data, associated with skewness, and the presence of outliers led to the choice of the interquartile range as the best and most feasible measure of variability [24]. The average brace wear per month, as recorded by the thermal sensor, was considered, and the consistency index was defined as the difference of at least one hour within the interquartile range [24]. This formula was used to define patients as either “inconsistent” or “consistent” in their brace wear, with respect to daily pattern compliance.

Short-term outcome was assessed by comparing the baseline Cobb angles with those in-brace and at the 6-month follow-up. Progression and improvement were defined as changes in the Cobb angle > 5° between two out-of-brace radiographs.

### 2.4. Statistical Analysis

Descriptive statistics were used to summarise key variables based on their distribution. Continuous variables were reported as means and standard deviations, whereas categorical variables were presented as counts and percentages within each diagnostic subgroup. Group comparisons for categorical variables were conducted using a chi-square test.

Adherence was analysed both as a continuous variable and as a categorical classification. For the continuous format, we reported the average percentage of brace wear time relative to the recommended duration. For the categorical format, patients were divided into three adherence groups [24]:

- Low—wore the brace on average below 70% of the recommended time;
- Medium—wore the brace between 70% and 89% of the recommended time;
- High—wore the brace 90% or more of the recommended time.

Binary logistic regression was used to identify predictors of brace-wearing consistency. Due to sample size constraints and the recommended ratio of events per variable, the base model included only a limited number of predictors. Five covariates were selected a priori based on clinical relevance and prior literature: Adherence, patient age, gender, initial Cobb angle, and brace type. To assess model robustness and potential confounding, a series of sensitivity analyses was conducted by sequentially adding related variables: recommended brace-wearing time, scoliosis type, primary curve location, and Risser sign. Each variable was entered individually into the base model to evaluate its incremental contribution and potential impact on existing predictors. Odds ratios (ORs) with 95% confidence intervals (CIs) were reported for all predictors. Statistical significance was set at *p* < 0.05. Model fit and predictor stability were assessed via Wald statistics and changes in coefficient estimates across models.

Receiver Operating Characteristic (ROC) analysis was performed to evaluate the discriminative ability of the adherence-based classification model.

A general linear model (GLM) was conducted to examine the effects of demographic and clinical predictors on short-term treatment outcome and in-brace correction. The dependent variable was the composite score of short-term therapeutic response. Independent variables included sex, scoliosis type, brace-wearing consistency, adherence category, age, and Risser stage, along with selected two-way interaction terms (sex × Adherence, sex × consistency, scoliosis type × consistency, and scoliosis type × Adherence). Type III sum of squares was used for hypothesis testing. Model fit was evaluated using the F-statistic, significance level (α = 0.05), and partial eta squared (η^2^) as a measure of effect size.

The association between brace adherence and clinical improvement was further evaluated via univariate binary logistic regression. Brace-wearing consistency (e.g., high vs. low/medium Adherence) served as the independent variable. Results were reported as odds ratios (ORs) with 95% confidence intervals (95% CI).

All statistical analyses were performed using IBM SPSS Statistics, version 30 (IBM Corp., Armonk, NY, USA).

## 3. Results

The study included 114 patients with IS (100 with AIS, 14 with JIS), for whom 6-month results were available, and data were complete (Table 1). Ninety-five were females, and nineteen were males, age of 12.7 ± 2.4 years and an initial Cobb angle of the major curve of 30.8 ± 10.4°.

Mean Adherence was 85.3 ± 18.9% in the whole population. A slightly higher Adherence was observed in the JIS group (92.8 ± 8.2%) than in the AIS group (84.2 ± 19.7%). When stratified by Adherence level, the majority of patients (58.8%) fell into the High-Adherence category, with JIS patients showing a notably higher proportion (71.4%) in the High-Adherence group and no cases in the Low-Adherence group. In contrast, 19.0% of AIS patients were classified as Low-Adherence. Stratification by sex revealed comparable Adherence rates between males (88.3 ± 19.1%) and females (84.7 ± 18.9%). The majority of both male (73.7%) and female (55.8%) patients were classified in the High-Adherence subgroup, with low Adherence observed in 15.8% of males and 16.8% of females (Table 2).

The chi-square test confirmed a significant association between scoliosis type (JIS vs. AIS) and consistency classification (χ^2^ = 6.84; df = 2; *p* = 0.033), indicating that patients with JIS were more likely to exhibit high Adherence in brace wear compared to those with AIS. The chi-square test did not confirm a significant association between sex (male vs. female) and adherence classification (χ^2^ = 2.70; df = 2; *p* = 0.280), indicating that adherence patterns in brace wear were comparable between male and female patients.

Consistency of brace wear differed significantly between diagnostic subgroups (Table 3). While only 19.3% of the total sample showed high consistency, the proportion was markedly higher in the JIS group (42.9%) than in the AIS group (16.0%).

A chi-square test confirmed a significant association between scoliosis type (JIS vs. AIS) and consistency classification (χ^2^ = 6.21; df = 1; *p* = 0.013), indicating that patients with JIS were more likely to exhibit high Adherence in brace wear compared to those with AIS.

Mean in-brace correction across the entire cohort was 52.5 ± 23.2%, with higher correction observed in the JIS subgroup (68.1 ± 26.1%) compared to AIS patients (50.3 ± 22.0%). Regarding short-term outcomes, 55% of patients improved, 42% remained stable, and only 3% worsened. Within subgroups, JIS patients demonstrated a more favourable distribution, with 64% showing improvement and no cases of deterioration. In contrast, 54% of AIS patients improved, 43% remained stable, and 3% worsened (Table 4).

An independent samples t-test revealed a significant difference in in-brace correction between groups by scoliosis type (JIS vs. AIS) (t = 2.759; df = 110; *p* = 0.007), indicating that the JIS group achieved higher correction percentages, with a mean difference of 17.8%. Fisher’s exact test did not confirm a statistically significant association between scoliosis type (JIS vs. AIS) and short-term outcome (Exact *p* = 0.718).

Short-term treatment outcomes were stratified according to brace wear consistency and adherence level (Table 5). Among participants with consistent brace wear, 64% showed improvement, compared to 53% in the inconsistent group. Stability was more frequent among inconsistent users (45%) than among consistent users (32%), while deterioration was rare across both groups (2–4%).

When analysed by adherence level, patients with high Adherence demonstrated the most favourable outcomes, with 63% showing improvement and only 4% experiencing deterioration. Medium Adherence was associated with a balanced distribution of stable (57%) and improved (43%) outcomes. In contrast, low Adherence was characterised by a predominance of stable outcomes (53%) and the lowest rate of improvement (47%), with no cases of deterioration observed.

Fisher’s exact test did not confirm a statistically significant association between consistency in brace wear and short-term outcome (*p* = 0.302). Although Fisher’s exact test did not confirm a statistically significant association between Adherence and short-term outcome (*p* = 0.150), the observed distribution—particularly the higher proportion of improvement among patients with high Adherence—may suggest a potential clinical impact warranting further investigation in larger studies (Table 5).

In the initial step, the logistic regression model with only the intercept revealed a significant baseline probability (B = −1.431; Wald = 36.344; *p* < 0.001), with an estimated probability of consistent brace wear of 23.9% (Exp(B) = 0.239). The Omnibus test of model coefficients indicated that the inclusion of predictors significantly improved model fit compared to the intercept-only model (χ^2^ = 44.682; df = 5; *p* < 0.001), suggesting that the predictors (Adherence, age, gender, initial Cobb angle, brace type) collectively contributed meaningfully to explaining the likelihood of consistency.

The full model demonstrated good fit to the data (−2 Log Likelihood = 67.157). Pseudo R^2^ indices indicated substantial explained variance: Cox & Snell R^2^ = 0.324 and Nagelkerke R^2^ = 0.519, implying that the model accounted for approximately 52% of the variability in consistent orthosis use.

Following the inclusion of predictors, the overall classification accuracy increased to 86.0%, compared to 80.7% in the intercept-only model. The model correctly classified 95.7% of participants with inconsistent orthosis use, while accuracy for consistent users was 45.5%. This discrepancy highlights the model’s stronger predictive capacity in identifying non-adherent behaviour.

In the multivariable logistic regression model, the only statistically significant predictor of consistent orthosis use was patient Adherence (B = 0.413; Wald = 10.806; *p* = 0.001). A higher Adherence was associated with a 51.1% increase in the likelihood of consistent behaviour (Exp(B) = 1.511; 95% CI: 1.181–1.933). Other predictors—including age, sex, initial Cobb angle, and brace type—did not reach statistical significance (*p* > 0.05). However, brace type demonstrated borderline significance (*p* = 0.090) and a potentially clinically relevant effect (Exp(B) = 0.123), warranting further investigation (Table 6).

To assess the robustness of the baseline logistic model, a sensitivity analysis was conducted by sequentially including four additional variables: recommended brace wear time, scoliosis type, location of the primary curve, and Risser sign (Table 7). These variables were initially excluded due to limitations related to the events-per-variable (EPV) ratio, yet they are clinically relevant and frequently discussed in the literature.

Across all extended models, patient Adherence remained the dominant predictor of orthosis wear consistency, retaining statistical significance and a stable direction of effect (OR 1.51–1.62; *p* < 0.01). Other baseline variables—age, sex, and initial Cobb angle—were consistently non-significant, with minimal changes in coefficient estimates (Table 6).

Brace type (Cheneau vs. Sforzesco) showed borderline significance in the baseline model (B = −2.097; *p* = 0.090) and Models 1 and 3 (*p* ≈ 0.09), suggesting a potentially relevant effect. The negative coefficient indicates a reduced likelihood of consistent wear among Cheneau users, independent of Adherence. Although not statistically significant (*p* < 0.05), its consistent trend across models warrants further evaluation.

Among additional variables, scoliosis type showed borderline significance in Model 2 (B = −2.609; *p* = 0.074), while recommended wear time, curve localisation, and Risser sign remained non-significant (*p* > 0.18; Risser *p* = 0.894).

The sensitivity analysis confirmed the baseline model’s robustness, indicating that added variables did not alter core conclusions. Adherence remains the key behavioural predictor, with brace type and scoliosis type emerging as secondary factors in future research.

**Figure 3:**
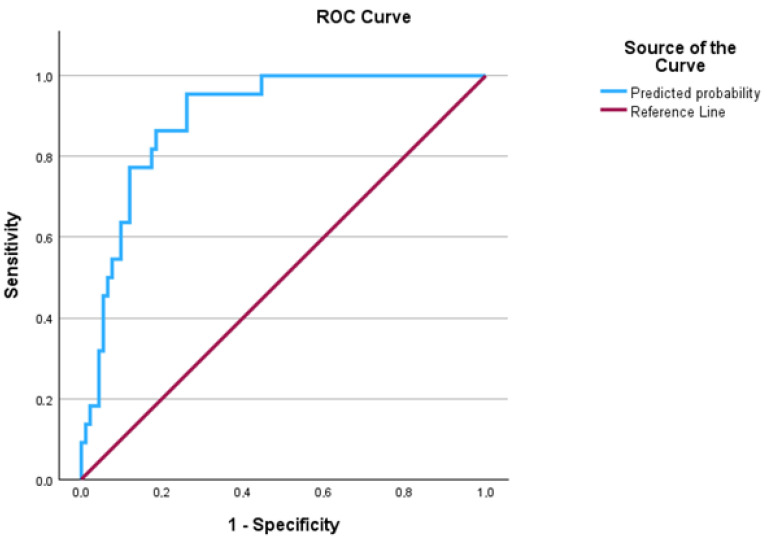
ROC curve for predicting consistency of brace use based on the Adherence model.

Receiver Operating Characteristic (ROC) analysis demonstrated excellent discriminative ability of the model in classifying participants based on orthosis adherence, with an Area Under the Curve (AUC) of 0.908 (SE = 0.030; 95% CI: 0.849–0.967; *p* < 0.001). This value indicates that the model can accurately distinguish between consistent and inconsistent users, underscoring its clinical utility (Figure 3).

The ROC analysis identified an optimal cut-off value of 0.226, at which the model achieved a sensitivity of 95.5% and specificity of 79.3%, yielding a maximum Youden index of 0.748. This threshold represents the most efficient point for classifying participants according to consistency, balancing accuracy across both categories.

Generalised linear models were conducted to evaluate the influence of demographic and clinical predictors on the percentage of in-brace correction and short-term outcome. The models included sex, scoliosis type, brace-wearing consistency, Adherence category, age, and Risser stage, along with selected two-way interaction terms (sex × Adherence, sex × Consistency, scoliosis type × Consistency, and scoliosis type × Adherence).

The overall models approached statistical significance:

- For in-brace correction: (F(12, 99) = 1.711; *p* = 0.076), explaining 17.2% of the variance, (R^2^ = 0.172; Adjusted R^2^ = 0.071);
- For Short-term treatment outcome: (F(12, 101) = 2.044, *p* = 0.028), explaining 19.5% of the variance (R^2^ = 0.195; Adjusted R^2^ = 0.100).

None of the individual predictors of in-brace correction reached statistical significance at the 0.05 level. The most notable effects were observed for age (F = 1.911; *p* = 0.170; η^2^ = 0.019) and Risser stage (F = 1.017; *p* = 0.316; η^2^ = 0.010), suggesting a potential trend toward better correction in younger patients with lower skeletal maturity. Interaction terms, including scoliosis type × Adherence category (F = 1.656; *p* = 0.201; η^2^ = 0.016), did not reach significance but may indicate differential effects across subgroups (Table 7).

Among individual predictors of short-term outcome, Adherence category (F = 3.300; *p* = 0.041; η^2^ = 0.061), and Risser stage (F = 4.298; *p* = 0.041; η^2^ = 0.041) demonstrated statistically significant effects. The high Adherence category was associated with more favourable short-term treatment outcomes, emphasising its role as a key predictor in therapeutic response. Risser stage also showed a significant effect, with lower skeletal maturity may have greater potential for correction. The interaction between scoliosis type and cooperation category approached statistical significance (F = 3.114; *p* = 0.081; η^2^ = 0.030), suggesting a differential effect of cooperation depending on deformity aetiology. Other predictors and interactions did not reach statistical significance (*p* > 0.05) (Table 8).

## 4. Discussion

In this paper, we reported Adherence and consistent wearing, and the results of a brace and exercise treatment for scoliosis, in a real clinical setting where thermal sensors supported patients. Monitoring the objective use of braces has proved helpful for patients, enabling the treating team to share data and helping them develop an objective understanding of how they manage their treatment. This approach allows for counselling and may help increase Adherence [16]. While several papers reported on the experimental use of thermal sensors [6,14,15], data from real-world clinical practice are scarce. Despite the availability of thermal sensors, these tools are not commonly used in clinical practice, and we found only a few papers reporting their use. The first paper reporting data from a cohort of patients using similar tools proved that compliance can be much higher than previously thought [25]. The objective compliance reported by Donzelli et al. was about 92% (95% CI 56.6–101.7), corresponding to more than 20 h per day in the IS patient cohort, versus a self-reported 100% (95% CI 70.7–100). Our results were not far off, with an average of 85.3 ± 18.9% across the whole population. In the consistent group, it was 98.6 ± 2.4%, while in the non-consistent group, it was 82.1 ± 19.7%. A retrospective study prescribed wearing a Boston brace for more than 20 h per day [26]. The overall measured wearing time was 16.9 ± 6.8. The mean bracing time at 6 months was 14.0 ± 8.0 h per day in the progressed group, compared with 17.4 ± 6.4 h per day in the non-progressed group. We calculated an overall Adherence of 84.5%, 70% in the progressed group, and 87% in the non-progressed group. These results are not far from ours.

Experimental studies reported an average Adherence far below these values. A small study by Helfenstein et al. reported an average adherence rate of 67.5% (range 19.0% to 97.1%) in 9 girls with AIS treated with the Cheneau brace [27]. Rahman applied thermal sensors to the braces of 55 girls and found an average adherence of 69.9 ± 31.5% [28]. They found no impact of the prescribed dosage, which ranged from 8 to 24 h per day, on Adherence. An experimental study by Nicholson et al. reported an average adherence of 65%, ranging from 8% to 90% [14]. In a prospective study, Katz et al. reported an overall Adherence rate of 35% in a group of patients prescribed 16 h per day and 27% in another group prescribed 23 h per day [7]. In the BrAIST study, the prescribed dosage was 18 (range, 0–23.0) [6]. This allows us to calculate an average Adherence of 67.2%. A pilot study of 28 patients prescribed 23 h per day reported a mean measured time of 14.1 ± 2.9 h [29]. The mean Adherence was 61.3 ± 12.6%. Other well-designed studies, such as the CONTRAIS, did not use sensors, so we cannot compare our findings [30].

In our study, the higher the prescribed dosage, the higher the Adherence, consistent with Donzelli [25] but in contrast to Katz [7]. In our study, patients with more severe curves were prescribed higher dosages and the Sforzesco brace. Those with less severe curves require fewer hours and a Cheneau brace. We do not know whether the different brace had any impact on Adherence. In the paper by Donzelli, the braces were more similar, being the Sforzesco and the Sibilla, both of which are quite symmetric [18]. In our case, we had a symmetric and an asymmetric one.

Excluding Donzelli [25], the best reported real-world average Adherence values from observational studies were 84.5% [26] and 78% [31]. Other authors reported compliance ranges of 33% [11] and 47% [32]. In a couple of studies, maximum values of 90% [14] and 97% [25] have been reported, while the median in this study was 91.7%. Notably, higher Adherence was reported in European studies (our own and Donzelli [25]), whereas studies reporting on North American patients showed worse results. This suggests that there may be cultural differences affecting Adherence and brace treatment. Another perspective is that higher Adherence was achieved in everyday clinical settings than in experimental studies. A possible explanation is that for such a demanding treatment, patients do not like to be part of research and have their treatment managed accordingly. This is consistent with the issues connected to running RCTs in the field. The BrAIST study was only partially randomised, with half of the patients deciding whether to be braced [6]. Bunge et al. failed to recruit patients for another RCT on bracing for scoliosis [33]. Coillard et al. reported an extremely high drop-out rate in the observed group of their RCT on SpineCor [34]. The authors of the CONTRAIS study needed 5 years to recruit only 135 patients, and 33% of patients declined to participate [30].

All patients in the consistent group also showed high Adherence, while 49% of those in the inconsistent group did. Adherence and consistency are directly correlated, even if sometimes patients who were not consistent proved to be adherent. In the overall sample, improvement was observed in 55% of patients, while deterioration was rare (3%). The JIS group demonstrated a higher mean in-brace correction (68% compared to 50% in AIS) and a greater proportion of improvement (64% versus 54%). When outcomes are considered in relation to consistency, improvement was achieved in 64% of patients with consistent brace wear, compared to 53% among those with inconsistent use. Similarly, improvement was observed in 63% of patients with high Adherence, whereas patients with medium or low Adherence showed lower rates of improvement (43–47%), with a predominance of stable outcomes. This synthesis of Table 1, Table 2 and Table 3 highlights that greater consistency and Adherence are associated with better correction and a higher proportion of improvement, particularly in the JIS group.

Usually, the best results are achieved in experimental studies rather than in everyday clinical practice. Here, things are different, and researchers should probably work more on large clinical databases to better align with clinical practice. There are several factors influencing Adherence and good results of conservative management of scoliosis during growth. A recent paper found that patients with high self-esteem, above-average peer relationships, and poor brace-specific attitudes have lower brace compliance. In comparison, patients with increased loneliness and parental religiousness have higher compliance [8]. The authors found no significant impact of body image, socioeconomic status, family dynamics, or school performance on brace use. These elements are mainly social and personal. Other studies investigated other factors. A recent paper focused on geographical factors. The authors found that living in the North of Italy, longer hours of brace prescription, female gender, younger age, and a lower Risser sign were associated with greater Adherence to brace wear [35]. Another study found only female gender and younger age to be predictive of good Adherence [36]. These factors can at least partially explain differences in Adherence between experimental and observational studies.

There are some limitations in our study. Our results reflect only short-term outcomes, which could enhance Adherence, while additional difficulties may arise over time. Nevertheless, most studies on compliance monitoring for bracing are also short-term. Another limitation is the retrospective design, which could lead to a selection bias and insufficient control of external variables (e.g., individual patient motivation). Nevertheless, we selected all consecutive patients with a thermal sensor installed in their brace, and there was no drop-out. This should mitigate the bias. Despite these limitations, the strengths of this paper include its focus on the everyday clinical setting, which increases the likelihood of generalisability.

## 5. Conclusions

In conclusion, we found that Adherence to brace treatment in scoliosis in everyday clinical practice can be higher than reported in most of the previous literature. This is based on a comprehensive team approach to support the young patients with scoliosis. Higher Adherence is connected to better results. Further studies based on larger samples should confirm our findings and describe the persistence of good Adherence over time. Further data are also needed for consistent wearing. Specifically, a prospective study with longer follow-up would allow for more valid conclusions about the impact of sensor-based monitoring on long-term outcomes.

## Figures and Tables

**Table 1:** Basic patient characteristics.

	All Patients		JIS		AIS	
	M ± SD	Min–Max	M ± SD	Min–Max	M ± SD	Min–Max
Age	12.7 ± 2.4	7 to 16	7.8 ± 0.9	7 to 9	13.4 ± 1.7	10 to 16
Cobb	30.8 ± 10.4	16 to 57	25.3 ± 10.2	16 to 47	31.6 ± 10.2	17 to 57
Risser	1.4 ± 1.4	0 to 4	0	0	1.6 ± 1.4	0 to 4

JIS—juvenile idiopathic scoliosis; AIS—adolescent idiopathic scoliosis; M ± SD—mean ± standard deviation; min–max—minimum to maximum value.

**Table 2:** Overall adherence rates and distribution across adherence subgroups in all patients, stratified by scoliosis type and sex.

		Scoliosis by Age		Gender	
	All Patients	JIS	AIS	Male	Female
Adherence (%)	85.3 ± 18.9	92.8 ± 8.2	84.2 ± 19.7	88.3 ± 19.1	84.7 ± 18.9
Adherence (subgroups)					
Low	19 (16.7%)	0 (0%)	19 (19.0%)	3 (15.8%)	16 (16.8%)
Medium	28 (24.6%)	4 (28.6%)	24 (24.0%)	2 (10.5%)	26 (27.4%)
High	67 (58.8%)	10 (71.4%)	57 (57.0%)	14 (73.7%)	53 (55.8%)

JIS—juvenile idiopathic scoliosis; AIS—adolescent idiopathic scoliosis. Results are presented as Mean ± SD and as frequency (%).

**Table 3:** Consistency of brace wear across diagnostic subgroups.

	All Patients	JIS	AIS
Consistency			
Low	92 (80.7%)	8 (57.1%)	84 (84.0%)
High	22 (19.3%)	6 (42.9%)	16 (16.0%)

AIS—adolescent idiopathic scoliosis; JIS—juvenile idiopathic scoliosis. Results are presented as frequency (%).

**Table 4:** In-brace correction and short-term outcome across diagnostic subgroups.

	All Patients	JIS	AIS
In-brace correction (%)	52.5 ± 23.2	68.1 ± 26.1	50.3 ± 22.0
Short-term outcome			
Worsened	3 (3%)	0 (0%)	3 (3%)
Stable	48 (42%)	5 (36%)	43 (43%)
Improved	63 (55%)	9 (64%)	54 (54%)

AIS—adolescent idiopathic scoliosis; JIS—juvenile idiopathic scoliosis. Results are presented as Mean ± SD and as frequency (%).

**Table 5:** Short-term outcome according to consistency in brace wear and adherence level.

	Consistency in Brace Wear		Adherence		
Short-Term Outcome	Inconsistent	Consistent	Low	Medium	High
Worsened	2 (2%)	1 (4%)	0 (0%)	0 (0%)	3 (4%)
Stable	41 (45%)	7 (32%)	10 (53%)	16 (57%)	22 (33%)
Improved	49 (53%)	14 (64%)	9 (47%)	12 (43%)	42 (63%)

Results are presented as frequency (%).

**Table 6:** Predictors of consistent brace wearing: results of multivariate logistic regression.

Variable	B	S.E.	Wald	*p*-Value	Exp(B)	95% CI for B
Adherence	0.413	0.126	10.806	0.001	1.511	1.181–1.933
Age	−0.221	0.157	1.995	0.158	0.802	0.590–1.089
Gender	−0.692	0.803	0.743	0.389	0.500	0.104–2.415
Initial Cobb angle	−0.002	0.052	0.002	0.969	0.998	0.901–1.106
Brace type	−2.097	1.238	2.868	0.090	0.123	0.011–1.391
Constant	−36.102	11.974	9.090	0.003	—	—

B—regression coefficient; S.E.—standard error; Wald—Wald chi-square test statistic; *p*-value—probability value indicating level of statistical significance; Exp(B)—exponentiated regression coefficient (odds ratio); 95% CI for B—95% confidence interval for the regression coefficient.

**Table 7:** Sensitivity analyses of logistic regression models predicting consistency.

Variable	Base Model	Model 1:	Model 2: +Scoliosis Type B (*p*)	Model 3: +Primary Curve Location B (*p*)	Model 4: +Risser Sign
	B (*p*)	+Recommended Wearing Time B (*p*)			B (*p*)
Adherence	0.413 (0.001)	0.444 (0.001)	0.466 (0.001)	0.481 (0.001)	0.480 (0.001)
Age	−0.221 (0.158)	−0.301 (0.100)	0.013 (0.958)	−0.054 (0.837)	−0.031 (0.920)
Gender	−0.692 (0.389)	−0.897 (0.287)	−0.710 (0.413)	−0.794 (0.374)	−0.816 (0.369)
Initial Cobb angle	−0.002 (0.969)	−0.022 (0.708)	−0.040 (0.542)	−0.034 (0.607)	−0.031 (0.666)
Brace type	−2.097 (0.090)	−2.202 (0.093)	−2.160 (0.127)	−2.423 (0.097)	−2.351 (0.131)
Recommended wearing time		0.368 (0.350)	0.504 (0.232)	0.600 (0.180)	0.593 (0.188)
Scoliosis type		—	−2.609 (0.074)	−2.284 (0.131)	−2.341 (0.137)
Primary curve location		—	—	0.967 (0.298)	0.963 (0.301)
Primary curve location		—	—	0.563 (0.538)	0.563 (0.538)
Risser sign		—	—	—	−0.046 (0.894)

B—regression coefficient; *p*—probability value indicating statistical significance.

**Table 8:** Analysis of variance for in-brace correction and short-term treatment outcome based on clinical and behavioural predictors.

	In-Brace Correction				Short-Term Treatment Outcome		
Source	df	F	*p* Value	Partial η^2^	F	*p* Value	Partial η^2^
Intercept	1	21.711	<0.001	0.180	4.255	0.042	0.040
Gender	1	1.178	0.280	0.012	0.010	0.920	0.000
Scoliosis type	1	0.114	0.736	0.001	3.297	0.072	0.032
Consistency	1	0.137	0.713	0.001	0.002	0.965	0.000
Adherence category	2	0.122	0.885	0.002	3.300	0.041	0.061
Age	1	1.911	0.170	0.019	0.812	0.370	0.008
Risser	1	1.017	0.316	0.010	4.298	0.041	0.041
Gender × Adherence	2	0.097	0.908	0.002	0.267	0.766	0.005
Gender × Consistency	1	0.026	0.873	0.000	0.164	0.687	0.002
Scoliosis type × Consistency	1	0.118	0.732	0.001	0.365	0.547	0.004
Scoliosis type × Adherence	1	1.656	0.201	0.016	3.114	0.081	0.030
Model (overall)	12	1.711	0.076	0.172	2.044	0.028	0.195

df—degrees of freedom; F—F-test statistic; *p*-value—probability value indicating the level of statistical significance; partial η^2^ (partial eta squared)—measure of effect size representing the proportion of variance explained by the factor, controlling for other variables.

## Data Availability

The raw data supporting the conclusions of this article will be made available by the authors on request.

## Abbreviations

The following abbreviations are used in this manuscript:

AIS	Adolescent Idiopathic Scoliosis
AUC	Area Under the Curve
CI	Confidence Interval
CI	(Consistency Index) Consistency Index
GLM	General Linear Model
IQR	Interquartile Range
IS	Idiopathic Scoliosis
JIS	Juvenile Idiopathic Scoliosis
OR	Odds Ratio
PSSE	Physiotherapeutic Scoliosis Specific Exercises
ROC	Receiver Operating Characteristic
SD	Standard Deviation
TLSO	Thoraco-Lumbo-Sacral Orthosis
